# Effect of led photobiomodulation on analgesia during labor: Study protocol for a randomized clinical trial

**DOI:** 10.1097/MD.0000000000011120

**Published:** 2018-06-22

**Authors:** Maria Aparecida dos Santos Traverzim, Sergio Makabe, Daniela Fátima Teixeira Silva, Christiane Pavani, Sandra Kalil Bussadori, Kristianne Santos Porta Fernandes, Lara Jansiski Motta

**Affiliations:** a Post-graduate Program in Biophotonics Applied in Health Sciences of Nove de Julho University; b Medical Undergraduate Course at Nove de Julho University, São Paulo, Brazil.

**Keywords:** labor, light-emitting diode, pain, photobiomodulation

## Abstract

**Background:**

Labor pain is one of the most intense pains experienced by women, which leads to an increase in the number of women opting to undergo a cesarean delivery. Pharmacological and nonpharmacological analgesia methods are used to control labor pain. Epidural analgesia is the most commonly used pharmacological analgesia method. However, it may have side effects on the fetus and the mother. Light-emitting diode (LED) photobiomodulation is an effective and noninvasive alternative to pharmacological methods.

**Objectives:**

To evaluate the effects of LED photobiomodulation on analgesia during labor.

**Methods:**

In total, 60 women in labor admitted to a public maternity hospital will be selected for a randomized controlled trial. The participants will be randomized into 2 groups: intervention group [analgesia with LED therapy (n = 30)] and control group [analgesia with bath therapy (n = 30)]. The perception of pain will be assessed using the visual analogue scale (VAS), with a score from 0 to 10 at baseline, that is, before the intervention. In both the groups, the procedures will last 10 minutes and will be performed at 3 time points during labor: during cervical dilation of 4 to 5 cm, 6 to 7 cm, and 8 to 9 cm. At all 3 time points, pain perception will be evaluated using VAS shortly after the intervention. In addition, the evaluation of membrane characteristics (intact or damaged), heart rate, uterine dynamics, and cardiotocography will be performed at all time points.

**Expected outcomes:**

The use of LED photobiomodulation will have an analgesic effect superior to that of the bath therapy.

## 1 Introduction

Labor pain is one of the most severe and agonizing events in the life of a woman.^[1–3]^ In previous studies, approximately 41% of women have reported that the high level of pain during natural labor and the fear of pain were the most important reasons for choosing a cesarean section.^[4,5]^ Although pain is a common phenomenon during labor,^[6]^ analgesia for pain relief during labor may be necessary to ensure that labor occurs as naturally as possible,^[7]^ thus benefiting the fetus and the mother.

Labor pain can be controlled by pharmacological and nonpharmacological methods.^[1,4,6,7]^ The use of drugs is the most common method, particularly epidural analgesia.^[8,9]^ However, pharmacological methods may have side effects on the fetus and the mother.^[8,9]^

The administration of epidural analgesia interferes with the secretion of hormones involved in labor, such as oxytocin, prostaglandin-F2a, and β-endorphin;^[6,10,11]^ this interference increases the risk of side effects, such as hypotension, delays the progress of labor, and increases the need for cesarean delivery.^[6,12,13]^ The concern regarding the side effects of pharmacological methods and the philosophy of encouraging vaginal delivery have promoted the discussion pertaining to the existing nonpharmacological methods.

Nonpharmacological alternatives that have been shown to be effective in pain relief include Swiss ball exercises and the hot bath therapy.^[14–18]^ The authors of a systematic review have reported that the hot bath therapy reduced pain in women with a cervical dilation of 8 to 9 cm and therefore reduced the need for pharmacological analgesia.^[14,19]^ The authors emphasized that nonpharmacological methods are beneficial and presented few side effects and contraindications. Furthermore, nonpharmacological interventions are noninvasive and favor the active participation of the mother during labor.^[14,19]^

Therefore, finding analgesic alternatives using effective nonpharmacological methods is an interesting challenge. Photobiomodulation is a noninvasive nonpharmacological intervention used for pain management in different areas of health sciences, such as orthopedics, physical therapeutics, dentistry, etc.^[20–23]^ Photobiomodulation therapy comprises the use of radiation sources, such as laser and light-emitting diodes (LEDs), for the application of nonionizing light; these radiations produce physiological benefits, such as increased microcirculation and ATP synthesis and decreased reactive oxygen species production.^[20–23]^

The analgesic effect of photobiomodulation occurs through the reabsorption of exudates, the elimination of algogenic substances, and via changes in the electrical conduction of the stimulus by maintaining the ionic gradient on both sides of the cell membrane, thereby avoiding or reducing depolarization. The effects of LED therapy on pain management, including the increase in blood flow and relaxation of spasms, have attracted the attention of professionals from different health sectors.^[24]^

The use of LED improves acute orthopedic conditions, such as sprains,^[24–26]^ postsurgical pain, cervical injury,^[27]^ upper and lower back pain, neck pain, and fibromyalgia.^[27–32]^ Because of the broad effects of photobiomodulation, LED therapy may be used as a nonpharmacological and noninvasive alternative for managing pain during labor and may provide comfort to the woman without producing any side effects.

The objective of this protocol is to evaluate the effect of LED photobiomodulation on pain management during natural labor compared with that of bath therapy. The second objective is to evaluate the fetal conditions at birth and in the first hours of life following LED photobiomodulation.

## 2 Methods

### 2.1 Study design

This randomized controlled trial will be conducted at the maternity ward of a public hospital in São Paulo, state of São Paulo, Brazil. In total, 60 uniparous or multiparous women who request analgesia during labor will participate in the study.

This study complies with the ethical guidelines in human research and was approved by the Research Ethics Committee of the Mandaqui Hospital (São Paulo, Brazil) under Opinion number 2.47.650 and has been registered in Clinical Trials under Protocol No NCT03496857. This is a randomized controlled clinical study; for the purpose of greater transparency and quality of the research, Table 1 summarizes the enrollment, intervention, and assessment schedule, all of which are in accordance with the SPIRIT (Standard Protocol Items: Recommendations for Interventional Trials) recommendations.

**Table 1:**
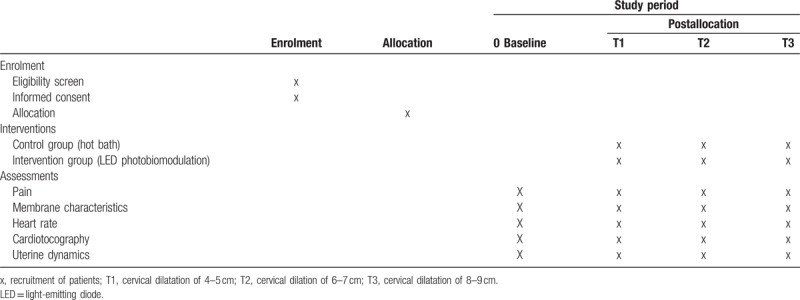
Schedule of enrolment, interventions, and assessments of treatments.

The participants will be randomized into 2 groups: intervention group [analgesia with LED therapy (n = 30)] and control group [analgesia with bath therapy (n = 30)]. The perception of pain will be evaluated using the visual analogue scale (VAS) with a score from 0 to 10 at baseline, that is, before the intervention. In both the groups, the procedure will last 10 minutes at each of 3 time points during labor: cervical dilation of 4 to 5 cm, 6 to 7 cm, and 8 to 9 cm.^[33]^ At the 3 time points, the level of pain will be assessed using VAS shortly after the intervention. Pharmacological analgesia will be promptly performed whenever necessary; in these cases, the participant will be excluded from the study and replaced with another consecutive participant.

### 2.2 Objectives of the study

Primary objective: To evaluate the effect of LED photobiomodulation on analgesia during labor as an alternative nonpharmacological intervention.

Research hypotheses:

Hypothesis 0 (null): the use of LED photobiomodulation exerts an analgesic effect similar to that of the bath therapy.

Hypothesis 1: the use of LED photobiomodulation exerts an analgesic effect superior to that of the bath therapy.

Hypothesis 2: the use of LED photobiomodulation exerts an analgesic effect inferior to that of the bath therapy.

Secondary objective: To evaluate fetal conditions using cardiotocography and the Apgar score in the first and fifth minutes after birth, and the level of satisfaction with the experience of childbirth using a questionnaire.

### 2.3 Participants

The study participants will be women aged >18 years, who are expected go into natural labor in the Maternity Hospital of Mandaqui. The inclusion criteria for the study will be as follows: women who request analgesia during labor; nulliparous and multiparous women; women with term gestation; women without previous diseases, including diabetes, neurological diseases, and metabolic diseases; and women in whom it is possible to evaluate fetal vitality during analgesia.

The exclusion criteria will be as follows: women whose labor is induced with medications; women who request drug analgesia during labor; and women who undergo cesarean delivery.

The participants will be recruited on admission at the maternity hospital and will be randomized in the 2 study groups after clarifying regarding the study objectives and obtaining their informed consent.

The sample size has been estimated using the difference in means in the score of the VAS from a study that evaluated analgesia during labor.^[34]^ The difference in the mean VAS score between the groups was set as 2 (standard deviation: 1.6). The minimum number of participants considering a power of 80% and level of significance of 5% was 28, and the minimum number of participants per group considering possible exclusions was 30.

### 2.4 Randomization

The patients will be distributed into the 2 groups by performing block randomization (n = 6) at a ratio of 1:1 according to a code generated by the SPSS software version 20.0 for Windows (IBM Corporation, Armonk, NY).

### 2.5 Procedures

#### 2.5.1 Intervention group: LED therapy

LED therapy sessions will be held in the prelabor room. The patient who will undergo analgesia and the professional responsible for placing the LED plate on the patient's back, between T10 and L2, will be present at the time of the intervention. The LED plate will be covered with clear disposable polyvinyl chloride to avoid cross-contamination and ensure hygiene. During the interventions, the patient will be allowed to choose the position that is the most comfortable for her.

Three 10-minute LED applications will be performed when the patient has a cervical dilatation of 4–5, 6–7, and 8–9 cm. Data on the level of pain, characteristics of the membrane (intact or damaged), heart rate, cardiotocography, and uterine dynamics will be collected after each intervention.

The LED device used is SportLux (Cosmedical, São Paulo, Brazil), and the dose parameters are shown in Table 2.

**Table 2:**
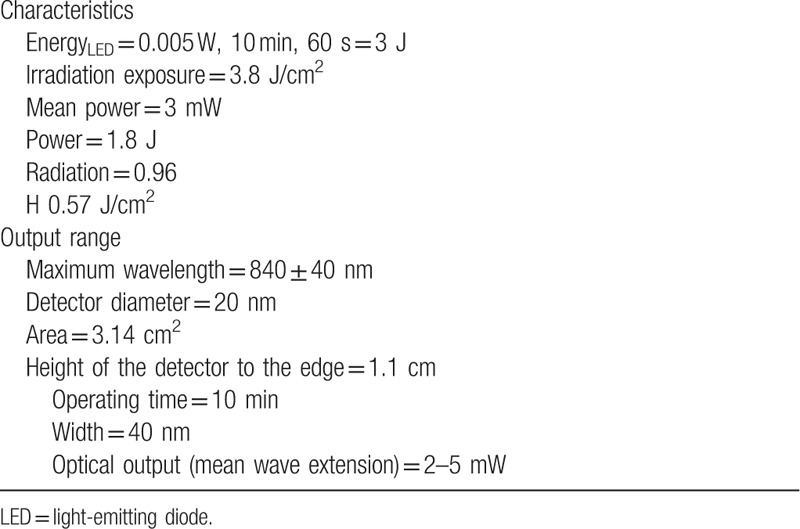
Dose parameters used in the protocol of the application of analgesia with light-emitting diode therapy.

### 2.6 Control group: bath therapy

The method of analgesia with the bath therapy will be performed using a hot shower at 37°C for 10 minutes. After showering the entire body or the back for 5 minutes, the participants will be allowed to direct the water flow to any area of the body that feels the most comfortable and to adjust the temperature themselves for improved comfort. Bath therapy will be performed at 3 time points during labor: at cervical dilatation of 4–5 cm, 6–7 cm, and 8–9 cm. Data on the level of pain, membrane characteristics (intact or damaged), heart rate, cardiotocography, and uterine dynamics will be collected after the bath therapy by performing the same measurements used in the intervention group.

### 2.7 Data analysis

The distribution of the VAS scores will be analyzed, and the continuous variables with normal data distribution will be compared using the *t* test and expressed as mean ± standard deviation. The categorical variables will be analyzed using the *χ*^2^ test or Fisher exact test. Non-normal data will be analyzed using the Mann–Whitney *U* test. All analyses will be performed using the statistical software SPSS version 20.0 for Windows (IBM Corporation, USA) at a level of significance of 5%.

## 3 Discussion

This study describes the protocol for a randomized controlled clinical trial aimed to evaluate the effect of LED therapy on analgesia during labor. The advantages of using LED therapy for analgesia during labor include the ease of application, the feasibility of application by the same team that assists the patient during labor, the possibility of the patient choosing the position that feels the most comfortable, even under analgesia, and improved mobility; the patient can remain in the upright position to help the fetal descent.

The main contribution of this clinical trial is the development of an analgesic intervention during labor that is effective and accessible for use in various public and private health services. The findings of this study may help develop protocols for analgesia during labor, thus allowing patients to not be afraid of pain and promote vaginal delivery.

This study will be the first to use photobiomodulation for analgesia during labor, and the results may help elucidate the correlation between phototherapy in pregnant women and fetal characteristics during therapy. In case of favorable outcomes, this approach can be used as a noninvasive and nonpharmacological alternative for analgesia during labor.

## Author contributions

**Conceptualization:** Maria Traverzim, Sergio Makabe, Sandra Bussadori, Kristianne Fernandes, Lara Jansiski Motta.

**Data curation:** Maria Traverzim, Christiane Pavani.

**Formal analysis:** Christiane Pavani, Kristianne Fernandes, Lara Jansiski Motta.

**Investigation:** Maria Traverzim.

**Methodology:** Sergio Makabe, Daniela Silva, Sandra Bussadori.

**Supervision:** Lara Jansiski Motta.
